# Sentiment analysis based on a social media customised dictionary

**DOI:** 10.1016/j.mex.2021.101449

**Published:** 2021-07-11

**Authors:** Milene Dias Almeida, Vinicius Mothé Maia, Roberto Tommasetti, Rodrigo de Oliveira Leite

**Affiliations:** a Federal University of Rio de Janeiro, Brazil; b COPPEAD Graduate School of Business, Federal University of Rio de Janeiro, Brazil

**Keywords:** Social media sentiment, Sentiment measurement, Behavioural finance, Sentiment perceptual map

## Abstract

This article presents a methodology to classify the polarity of words from selected Tweets. Usually, social media sentiment (SMS) is lexically determined, manually or by machine learning. However, these methods are either slow or based on a pre-established dictionary, thus not providing a customised analysis. We propose a methodology that, after having mined the topic-related Tweets, filters relevant words based on the mean and standard deviation frequency in positive and negative market days to remove neutral terms. Subsequently, through an ad hoc perceptual mapping, we assign a polarity to the dataset. This method allows the building of a dictionary associated with the investor sentiment customised to *that* organisation. A practical application was carried out to test the proposed methodology. The results were significant and in line with the behavioural finance theory, confirming that irrational investor feelings—expressed via social media—drive a portion of asset prices. Results also confirm the investor asymmetric behaviour under gain or loss scenarios, with the latter generating more impact than the former because people are risk-averse. The proposed method is expected to identify patterns of behaviour in social media linked to market oscillations, thereby contributing to risk management and optimising decision-making in the stock market.

• The use of both statistical and perceptual map filters allows a specific asset dictionary to be built;
• Textual sentiment analysis based on social media;
• The proposed method efficiently overcomes generic dictionaries and language issues.

## Graphical abstract

**Figure:**
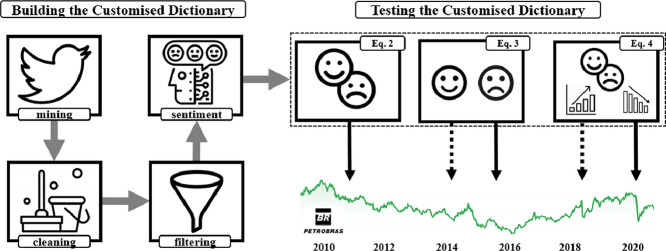

Specifications table

**Table:** 

Subject Area:	Economics and Finance
More specific subject area:	Behavioural finance Sentiment analysis
Method name:	Social Media Customised Dictionary
Name and reference of original method:	*N/A*
Resource availability:	Social Media Data; Python or R.

## Sentiment analysis background

The Efficient Market Hypothesis predicts asset prices fully reflecting all the available information and, accordingly, rational investors choose asset portfolios that diversify away from the idiosyncratic risk. As such, asset prices are only a function of market fundamentals. In contrast, behavioural finance theory suggests that irrational investor feelings drive a portion of asset prices [15,17]. In the context of the internet, social media (SM) represent a unique database of society's behaviour [4].

Studies differ as to how the SMS is estimated. Research related to textual sentiment analysis is mainly divided into both qualitative and quantitative approaches [11]. In the first category, feelings are classified manually in a lexical way, i.e. Loughran-McDonald Financial Dictionary [13]. In the second category, sentiment is classified by probabilistic approaches through machine learning, such as, for example, the Bayes Classification method [1].

Most studies use a proportional weighting of terms, considering all words in the list equally important. The Harvard-IV-4 open-domain,^1^ for instance, considers simple frequencies for words in the text that fall into each category. Other studies weigh each term differently [13] using two weighting schemes: a simple proportional weighting and another that weighs each word in proportion to the simple inverse frequency of the same word in the document. Thus, the more recurrent the term, the lower its weight, [14] calculating the value based on two levels: (i) word level, where the word receives a fixed value regardless of the context of the sentence in which the word appears; and (ii) sentence level, where the adverb influence determines a sentence value. The final value is the sum of the sentence value, and the relative value is the summed value divided by the number of sentences.

Two studies [11,12] found that a list of general words does not apply to financial texts. Thus, a specific dictionary models the SMS better than one adapted from other areas or than a generic one. Social media texts may contain opinions about several topics, but terms used to express opinions are usually specific and highly correlated to a particular domain [5]. Also, the list must be in the same language as the analysed text. A simple translation of a list of specific content into English could lead to an erroneous sentiment analysis result [2]. Finally, these investor sentiment measures currently show overall market sentiment rather than asset-specific sentiment [15].

Given the limitations of the previous literature discussed above, we propose a topic-customised dictionary from social media texts using a statistical technique and personalised perceptual mapping. Following the present study approach, the dictionary is formed by words “chosen” by the social media user-investors based on the stock return, without intervention by the researcher. At the same time, the application method is limited to listed companies with a unique name.

The novel contribution of this study is to customise the dictionary for individual organisations, filling the gaps in the existing dictionaries, which are generic and, thus, of lower quality. In this way, the proposed sentiment indicator can mitigate or enhance that particular firm's market movements.

## Building the customised dictionary

The construction of the sentiment indicator starts with mining for messages posted on social media containing the organisation's name, using a Twitter development account. As the texts are long, it is necessary to perform tokenisation, which converts each SM post into a unigram vector [12]. Then, the database needs to be cleaned by removing stop words with no semantic value.

The subsequent phase is to discard neutral words. These appear every day and at the same frequency regardless of the firm's daily stock price change. The filter excludes terms which show a frequency difference, in their mean and standard deviation, lower than 20% between positive and negative market days.

Thus, words positioned close to zero on the perceptual map were removed to determine the polarity of the terms. Multidimensional scaling is a method for visualising the degree of dissimilarity among objects. Based on these dissimilarity coefficients, a series of Euclidean distances is calculated. The data are usually plotted in two multidimensional Cartesian spaces [8]. Preliminarily it is necessary to calculate similarity across the set of objects to build a perceptual map. Suppose the words *a* and *b* receive similar values compared to other possible objects (*c, d, e*). In that case, multidimensional scaling attributes a lower Euclidean distance between *a* and *b* than that among the other objects. The axes represent the positive and negative poles so that the more extreme the word is, the more positive or negative this will be.

The classification approach used by this methodology is called the “bag of words”, which separates dictionary terms into positive and negative. Thus, words receive numerical scores according to their polarity, positive (+1) and negative (-1) [9,13].

This list of positive and negative words related to the company will compose the customised dictionary. The value of a Tweet sentiment is estimated based on the weight of the positive and negative words. This index can be applied, for example, to predict or explain stock price fluctuations in publicly traded companies. The daily sentiment variable is the average sentiment of Tweets on a given day. The measure is the total of positive words minus negative ones, divided by the total number of words [7], as follows in Eq. (1).

(1) $$ASE_{t}=PSE_{t}+NSE_{t}=\frac{\sum PWO_{t}}{\sum TWO_{t}}+\;\frac{\sum NWO_{t}}{\sum TWO_{t}}$$

Where:

$ASE_{t}$: Tweet's Average Sentiment;

$PSE_{t}$: Tweet's Positive Sentiment;

$NSE_{t}$: Tweet's Negative Sentiment (with its polarity, this is -1);

$PWO_{t}$: Number of Tweet's Positive Words;

$NWO_{t}$: Number of Tweet's Negative Words (with their polarity, this is -1);

## Method validation

Between January 1st, 2010 and June 30th, 2020, 3,826,463 Tweets were collected containing the search term “petrobras”. Petróleo Brasileiro S.A., better known as Petrobras, is a Brazilian state-owned multinational corporation operating in the petroleum industry. The company was ranked 120th in the most recent Fortune Global 500 list^2^ and is the 70th largest public company as per Forbes Global 2000.^3^ The authors decided not to use the Tweets containing the company ticker name (PETR3 and PETR4) since this sample was limited in quantity and quality.

After the collection, the data was cleaned, the words filtered, and the polarity was classified based on the perceptual mapping, building the sentiment indicator. This variable was included in Sharpe's model [16], which analyses the relationship between a stock price return and a single index. The return is the log change in daily Petrobras stock price [6], while the market index is the Bovespa. Regarding the sentiment, its first difference was used to capture the change in global sentiment instead of the absolute (positive or negative) status. For instance, if the index moves from 0.90 to 0.75, it continues to be positive in absolute terms, but its trend—which is our focus variable—is negative.

Finally, regressions were made. The linear regression model for time series was run with the estimation made by ordinary least squares (OLS). The following tests were carried out to identify potential residual distribution issues: Jarque-Bera test for Normality, Breusch-Pagan test for Heteroscedasticity and Ljung-Box test for autocorrelation. When one of the above tests rejects the residual distribution hypothesis, Autocorrelation-Consistent Standard Errors (HAC) was applied.

First, based on the market model developed by Sharpe [16], an equation was built to verify whether Petrobras’ return is related to the textual sentiment present in Tweets, in addition to the market return, proxied by the Ibovespa index, as per the following equation:

(2) $$FRE_{t}=\;\alpha +\;\beta_{1}MRE_{m,t}+\;\beta_{2}ASE_{t}+\;\varepsilon_{t}$$

Where:

$FRE_{t}$: Firm Return, as the logarithm of Petrobras daily price;

$MRE_{t}$: Market Return, as the logarithm of Bovespa Index daily change;

Secondly, the sentiment variable was split into positive and negative. This path was chosen since different behaviours between the Tweet's positive ($PSE_{t}$) and negative sentiment ($NSE_{t}$) is expected: positive for $\beta_{2}$, increasing the firm's return, and negative for $\beta_{3}$, decreasing the firm's return [9].

(3) $$FRE_{t}=\;\alpha +\;\beta_{1}MRE_{m,t}+\;\beta_{2}PSE_{t}+\beta_{3}NSE_{t}+\;\varepsilon_{t}$$

Finally, we investigate whether the sentiment amplifies or diminishes the (positive or negative) market returns. The aim is to determine if, when the market is pessimistic, the positive sentiment can moderate, smoothing the impact on the firm's return, while the negative can exacerbate the effect on the firm's return.

The opposite expectation is when the market is optimistic. Therefore, the expected $\beta_{3}$ sign is positive and $\beta_{4}$ is negative in the following equation.

(4) $$FRE_{t}=\;\alpha +\;\beta_{1}MRE_{m,t}+\;\beta_{2}ASE_{m,t}+\;\beta_{3}MPR_{m,t}*ASE_{t}+\;\beta_{4}MNR_{m,t}*ASE_{t}+\;\varepsilon_{t}$$

Where:

$MPR_{t}$: Market Positive Return, as the Bovespa Index positive daily log return;

$MNR_{t}$: Market Negative Return, as the of Bovespa Index negative daily log return.

The results of the regressions are shown in Table 1.

**Table 1:** Sentiment impact on Firm return (Petrobras) (2,550 Observations)

Regressors	Sign	Eq. (2)	Eq. (3)	Eq. (4)
MRE	+	1.457 *** (< 0.001)	1.453 *** (< 0.001)	1.432***(< 0.001)
ASE	+	1.579e-16 * (0.049)	-	4.759e-17 (0.276)
PSE	+	-	-3.436e-17 (0.318)	-
NSE	+	-	2.036e-16 * (0.026)	-
MPR*ASE	+	-	-	-8.054e-15 (0.257)
MNR*ASE	-	-	-	-4.911e-15*** (< 0.001)
α	na	-2.823e-04 (0.433)	-1.843e-04 (0.617)	-2.575e-04 (0.472)
Adjusted R^2^		0.618	0.620	0.627
Normality *p*-value		< 0.001	< 0.001	< 0.001
Heteroscedasticity *p*-value		< 0.001	< 0.001	0.898
Autocorrelation *p*-value		0.928	0.901	0.664

Normality: Jarque-Bera test; Heteroscedasticity: Breusch-Pagan test. Ljung-Box test for autocorrelation (10 lags). Estimation method: OLS with Autocorrelation-Consistent Standard Errors (HAC). *p < 0.10; ** 0.01 < *p* < 0.05; *** *p* < 0.01.

The findings are in line with the financial behaviour theory. The market return is significant and positive, demonstrating that variation of the SM textual sentiment is related to the return. These results corroborate studies that concluded that sentiments expressed via Twitter are related to market returns [3,15].

In Eq. (3), the variation in the negative sentiment is the only variable associated with the firm's return. This result confirms the theory by [10], which supports an asymmetry between gains and losses: people feel more sensitised by a loss than by a gain, even if it is comparable in magnitude. Similar results are reported by [9].

With Eq.( 4), the variation of the sentiment is significant, and its coefficient is negative in an adverse market scenario. The $\beta_{4}$ negative sign, in an adverse market, implies the moderating role of the sentiment variable ($ASE_{t}$). In a pessimistic market scenario, the negative firm's SM Average Sentiment exacerbates the impact on company stock prices, while the positive SM Average Sentiment smooths it.

Finally, Table 1 shows that the proportion of the variance in the dependent explained by the independent variables, represented by R^2^, increases from Eq. (2), where the average sentiment is the observed regressor, to Eq. (3), where the above regressor is split into positive and negative (sentiment), and finally to Eq. (4), where the same variable interacts with favourable and unfavourable market days. These results represent an additional confirmation of investor asymmetric behaviour under gain or loss scenarios, with the latter generating more impact than the former because people are risk-averse. It is a natural behaviour to defend ones assets [9,10].

## Conclusions

The above results show that it is possible to construct a customised dictionary from the SMS associated with each specific company. The process starts with mining Tweets related to the organisation, filtering relevant words based on the average and standard deviation of the frequency of terms in positive and negative market days, to remove neutral words and, subsequently, through perceptual mapping, to associate the polarity with the term's dataset.

Creating a customised dictionary explains gains or losses in the company's value. With the construction of these maps, a powerful tool for marketing management and analysing investor sentiment about the organisation can emerge. This method is expected to identify behavioural patterns linked to market oscillations, thereby contributing to risk management and optimising decision-making in the stock market. For future research, it is suggested: (i) to include a weighting of the terms; (ii) to create a market index, “consolidating” individual company SMS. As to practical implications, the Social Media Customised Dictionary could predict future assets and returns. Specifically, in terms of the pre-opening and post-closing of the market, selected Tweets could be used to run the proposed model to predict future gains. In a nutshell, Tweets posted after the market has closed might contribute positively to the stock price on the following day.

## Declaration of Competing Interest

The authors declare that they have no known competing financial interests or personal relationships that could have appeared to influence the work reported in this paper.

## Footnotes

1 https://www.wjh.harvard.edu/~inquirer/homecat.htm.

2 https://fortune.com/company/petrobras/global500/

3 https://www.forbes.com/companies/petrobras/?sh=605f08fe2dea
